# Natural Silkworm Cocoon-Derived Separator with Na-Ion De-Solvated Function for Sodium Metal Batteries

**DOI:** 10.3390/molecules29204813

**Published:** 2024-10-11

**Authors:** Zhaoyang Wang, Zihan Zhou, Xing Gao, Qian Liu, Jianzong Man, Fanghui Du, Fangyu Xiong

**Affiliations:** 1 Shandong Provincial Key Laboratory of Chemical Energy Storage and Novel Cell Technology, College of Chemistry Engineering, Liaocheng University, Liaocheng 252059, China; wzy9218@126.com (Z.W.); 13884971808@163.com (Z.Z.); 18764892075@163.com (X.G.); 13562862100@163.com (Q.L.); manjianzong@lcu.edu.cn (J.M.); dufanghui@lcu.edu.cn (F.D.); 2 College of Materials Science and Engineering, Chongqing University, Chongqing 400044, China; 3 Chongqing Institute of New Energy Storage Materials and Equipment, Chongqing 401135, China

**Keywords:** sodium metal battery, separator materials, natural silkworm cocoon, Na-ion de-solvated function, mechanistic analysis

## Abstract

The commercialization of sodium batteries faces many challenges, one of which is the lack of suitable high-quality separators. Herein, we presented a novel natural silkworm cocoon-derived separator (SCS) obtained from the cocoon inner membrane after a simple degumming process. A Na||Na symmetric cell assembled with this separator can be stably cycled for over 400 h under test conditions of 0.5 mA cm^−2^–0.5 mAh cm^−2^. Moreover, the Na||SCS||Na_3_V_2_(PO_4_)_3_ full cell exhibits an initial capacity of 79.3 mAh g^−1^ at 10 C and a capacity retention of 93.6% after 1000 cycles, which far exceeded the 57.5 mAh g^−1^ and 42.1% of the full cell using a commercial glass fiber separator (GFS). The structural origin of this excellent electrochemical performance lies in the fact that cationic functional groups (such as amino groups) on silkworm proteins can de-solvate Na-ions by anchoring the ClO_4_^−^ solvent sheath, thereby enhancing the transference number, transport kinetics and deposition/dissolution properties of Na-ions. In addition, the SCS has significantly better mechanical properties and thinness indexes than the commercial GFS, and, coupled with the advantages of being natural, cheap, non-polluting and degradable, it is expected to be used as a commercialized sodium battery separator material.

## 1. Introduction

The growing energy demand has greatly prompted researchers to explore electrochemical energy storage systems (EESs) with low-cost, high energy density and environmental friendliness characteristics [1,2]. Among all available EESs, sodium metal batteries (SMBs) are considered to be the most promising substitutes to Li-ion batteries (LIBs) owing to the following unique advantages [3]: (i) SMBs work similarly to LIBs, so the research experience with LIBs can be utilized to develop SMBs [4]. (ii) Sodium resources are abundant and evenly distributed worldwide, which leads to a potential low cost of sodium metal anodes (SMAs) [5]. (iii) Sodium metal anodes possess low redox potential (−2.71 V vs. the standard hydrogen potential), light weight (23 g mol^−1^) and high theoretical specific capacity (1166 mAh g^−1^), endowing SMBs with high working voltage and high energy density [6]. Despite the attractive advantages, the development of SMBs has encountered various bottlenecks, one of which is the lack of high-quality separators [7].

As an important component of battery, the separator, on the one hand, bears the task of isolating the flow of electrons between the cathode and anode within the battery; on the other hand, it is also responsible for allowing ions to pass through smoothly [8]. There is no doubt that the quality of the separator determines the final performance of the battery. Compared to Na-ion batteries, SMBs place a higher demand on the electrolyte wettability and uptake capability, thermal stability, mechanical strength and ion distribution regulation ability of the separator [9]. Currently, the commercial Na-ion battery utilizes an LIB separator, i.e., a polypropylene (PP) or polyethylene (PE) separator. However, due to the drawbacks such as inferior electrolyte wettability, poor thermal stability and low electrolyte uptake capability, PP/PE separators are unsuitable for SMBs [10]. Although glass fiber separators (GFSs) can solve the above problems and are therefore widely used in laboratory research, their excessive thickness and poor mechanical properties make them difficult to apply in industrial production [11,12].

Scientists have made tremendous efforts to develop high-quality separators for SMBs [13]. Among them, biomass-derived separators have gained increasing attention due to their environmental friendliness, renewability and resource abundance [14]. Casas et al. cross-linked carboxymethyl cellulose (CMC) and hydroxyethyl cellulose (HEC) to prepare membranes with large specific surface area [15]. The Na||Na_3_V_2_(PO_4_)_3_(NVP) battery assembled with this biomass membrane as a separator delivered a residual capacity of 74 mAh g^−1^ after 10 cycles at 0.1 C with a nearly 100% Coulombic efficiency, which were both higher than that of the battery using a commercial Whatman GFS (61 mAh g^−1^, 96%). Wang’s group used electrospinning technology to construct cellulose nanocrystals into flexible bifunctional separators and applied them to SMBs [16]. This kind of separator can not only promote the uniform deposition of Na-ions on the anode by regulating the ion flow and nucleation behavior, but can also take advantage of the mechanical strength to block the continuous vertical growth of sodium dendrites, thus avoiding the occurrence of short circuits. These studies confirm the potential of biomass-derived separators for SMB applications and inspire subsequent researchers to continue to delve deeper into the natural treasure trove.

The silkworm cocoon is an amazing biological material that is naturally porous and layered, and therefore has morphological similarities to battery separators. Based on this similarity, silkworm cocoons have been initially developed as separators for LIBs [17,18,19,20,21,22]. Pereira et al. tested the suitability of silkworm cocoons as LIBs separators in carbonate-based and trifluoromethylsulfonyl-based electrolytes, and the results prove that the former electrolyte is more suitable for LIBs using cocoon separators [17]. In view of the multilayer structure of cocoons, Guo et al. examined the electrochemical performance of sublayers at different positions in cocoons and after stacking, and the results show that the inner layer is more suitable as a separator for LIBs, and the multilayer structure is more beneficial to the electrochemical performance [18]. It is well known that micro-morphology is an important factor affecting the electrochemical performance of the separator, and in order to regulate the pore structure in cocoon-based separators, Reizabal et al. treated cocoons by a salting method [19]. The battery using a cocoon separator with a pore size range of 106–250 μm showed the best electrochemical performance, i.e., a discharge capacity of 66.9 mAh g^−1^ at 2 C and a residual capacity of 56.9 mAh g^−1^ after 55 cycles. In addition to this, the lyophilization [20] and plasma treatment [21] of silk have been used to optimize the morphology of cocoon-based separators with exciting progress.

In this paper, we reported a novel natural silkworm cocoon separator (SCS) for SMBs. The unique advantage of SCSs over commercially available GFSs is that they can anchor ClO_4_^−^ in the electrolyte by virtue of the abundant amino functional groups in the protein molecular chain, thus enabling Na-ions to exhibit superior transport kinetics. Benefiting from this, the Na||SCS||Na symmetric cell is able to cycle stably for more than 400 h at a current density of 0.5 mA cm^−2^ with an areal capacity of 0.5 mAh cm^−2^, which far exceeds the effective cycling time (~20 h) of the Na||GFS||Na symmetric cell under the same test conditions. In addition, the Na||SCSs||NVP full cell exhibits a discharge capacity of 79.3 mAh g^−1^ at 10 C with a capacity retention rate of 93.6% after 1000 cycles. In order to facilitate the reader to compare the differences in electrochemical performance between the present work and previous cocoon separator studies, we summarize these findings in Table S1.

## 2. Results

### 2.1. Electrochemical Performance

**Figure 1:**
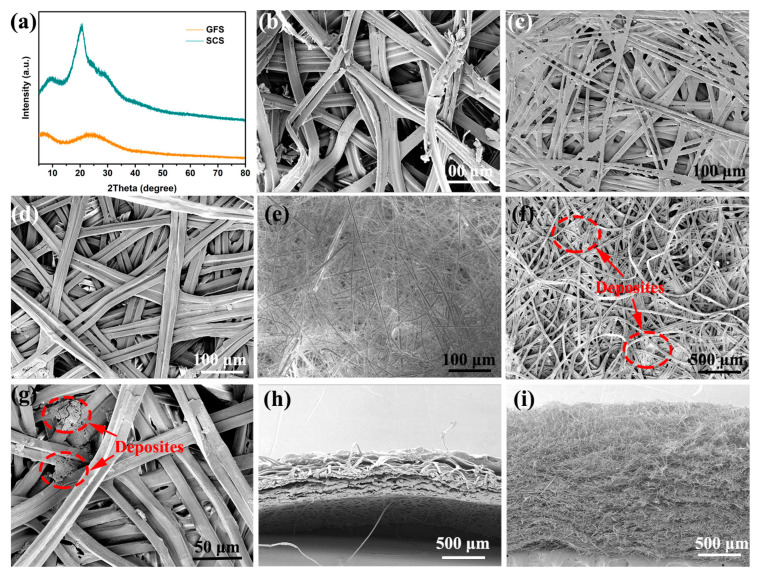
(**a**) XRD of the SCS and GFS. SEM top view images of the outer layer (**b**), pristine (**c**)/boiled (**d**) inner layers of the cocoon and the glass fiber separator (**e**). (**f**,**g**) SEM images of SCS samples after cyclic testing. SEM cross-section view images of the cocoon (**h**) and GFS (**i**).

In order to optimize the pore structure of silkworm cocoons and to expose the functional groups on their protein molecules, natural silkworm cocoons were boiled in a weak alkaline solution to remove the sericin [22]. The SCS sample has four broadened crystal diffraction peaks located at 9.1°, 20.5°, 23.0° and 29.4° (Figure 1a). These diffraction peaks should be attributed to the (100), (210), (002) and (300) crystal planes of β-sheet silkworm protein, respectively [23,24,25]. Table S2 summarizes the structural parameters of each grain surface calculated using Jade 6 software and the Debye–Scherrer formula, where a small grain size (<4 nm) implies the poor crystallinity of the SCS sample [26,27]. In contrast, no crystal diffraction peaks are observed in the XRD patterns of the GFS sample due to the disordered characteristics of glass fiber [28]. Scanning electron microscopy (SEM) was employed to observe the morphology features of the samples at the micron scale [29,30,31]. As shown in Figure 1b,c, the morphology of the inner and outer layers of the cocoon varies greatly: the silkworm in the outer layer of cocoon is fluffy and stacked with large pores; the silkworm in the inner layer of the cocoon is flat and stacked with small pores, and an obvious bonding phenomenon can be observed. This bonding phenomenon is significantly reduced after boiling (Figure 1d). In view of the advantages in regularity and pore structure, the inner layer of the cocoon was selected as the separator for SMBs. The GFS also exhibits a large pore structure and has no bonding points between its fibers (Figure 1e). The bonding between fibers is likely to be the structural root cause of the superior mechanical properties of the SCS over the GFS [32].

SEM images of the SCS samples after 100 cycles at 5 C show no significant changes in the SCS except that some deposits can be observed (as shown in Figure 1f,g). Based on previous studies, it is known that these sediments are metallic sodium crystals and sodium salt crystals, of which the former is predominant [33]. In order to evaluate the pore structure of the separators more comprehensively, SEM tests were performed on the cross-sections of the cocoons and GFS, respectively. As shown in Figure 1h, the cocoons were clearly stratified, with the inner layer being more regular and tightly packed compared to the outer layer, which is consistent with the results observed in Figure 1b,c. The SCS, taken from the inner layer of the cocoon, is much lower than the GFS (Figure 1i) in both porosity and tortuosity.

**Figure 2:**
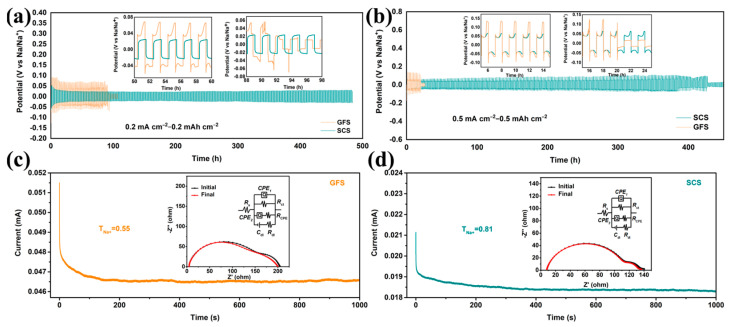
Characterization of sodium plating/stripping behavior in symmetric batteries. Voltage profiles of the symmetrical Na||GFS||Na and Na||SCS||Na cells under current areal capacities of (**a**) 0.2 mAh cm^−2^ and (**b**) 0.5 mAh cm^−2^. The *i*–*t* curves of Na||GFS||Na (**c**) and Na||SCS||Na (**d**) cells; the insets are the corresponding EIS curves before and after chronoamperometry test.

The Na||Cu asymmetric and Na||Na symmetric cells using SCSs and GFSs as separators were assembled for testing. As shown in Figure S1, the assembled Na||SCS||Cu battery shows a lower nucleation over-potential of 0.065 V than that of the Na||GFS||Cu battery (0.113 V), indicating that the SCS could decrease the nucleation resistance and local current density during Na deposition [34]. Figure 2 presents the voltage–time profiles of Na||Na symmetric cells with the SCS and the GFS. Notably, the Na||SCS||Na symmetric cell can stably cycle for ~480 h and the polarization voltage is stabilized at only 0.04 V throughout under 0.2 mA cm^−2^–0.2 mAh cm^−2^ test conditions (Figure 2a). As for the Na||GFS||Na battery, the polarization voltage under the same test conditions is close to 0.075 V and short circuit occurs when the battery run for about 100 h. At the test conditions of 0.5 mA cm^−2^–0.5 mAh cm^−2^, the Na||SCS||Na symmetric cell exhibits a polarization voltage of ~0.05 V (Figure 2b). After 400 h of steady operation, the polarization voltage of this battery rises slightly to ~0.09 V and eventually fails completely after 427 h of operation. As a comparison, the Na||GFS||Na cell displays an initial polarization voltage as high as 0.125 V and only stably cycles for about 20 h. The above results indicate that SCS is more beneficial as a battery separator for Na-ion transport and deposition/dissolution [35].

To clarify the effect of the SCS on Na-ion transport in batteries, chronoamperometry (CA) and corresponding electrochemical impedance spectroscopy (EIS) tests were performed on the Na||GFS||Na and Na||SCS||Na cells. After careful comparison, all the EIS curves in Figure 2c,d are well matched to the “*R*_s_(*QR*_ct_(*QR*_CPE_)(*C*_dl_*R*_dl_))” equivalent circuit with errors of less than 4.1%. Here, *Q* represents the constant phase element (CPE); *C*_dl_ represents double layer capacitance; and *R*_s_, *R*_ct_, *R*_CPE_ and *R*_dl_ represent solution impedance, charge transfer impedance, CPE impedance and double capacitance impedance, respectively [36,37]. As reported by the ZSimpwin 3.60 software fitting parameter (Table S3), the initial *R*_ct_ of the Na||SCS||Na cell (134.6 Ω) is much smaller than that of the Na||GFS||Na cell (205.7 Ω), indicating that the interfacial impedance between the SCS and the sodium metal anode is smaller. The Na^+^ transference number (*T*_Na+_) of the above symmetric cell can be calculated using the following equation [38,39]:

*T*_Na+_ = (*I*_s_ × (∆*V* − *I*_0_ × *R*_0_))/(*I*_0_ × (∆*V* − *I*_s_ × *R*_s_))

where ∆*V*, *I*_0_, *I*_s_, *R*_0_ and *R*_s_ are the constant applied voltage (10 mV), initial and steady-state currents, and the *R*_ct_ before and after the CA test. The calculated *T*_Na+_ of the Na||SCS||Na cell is 0.81, which is higher than the 0.55 of Na||GFS||Na cell.

**Figure 3:**
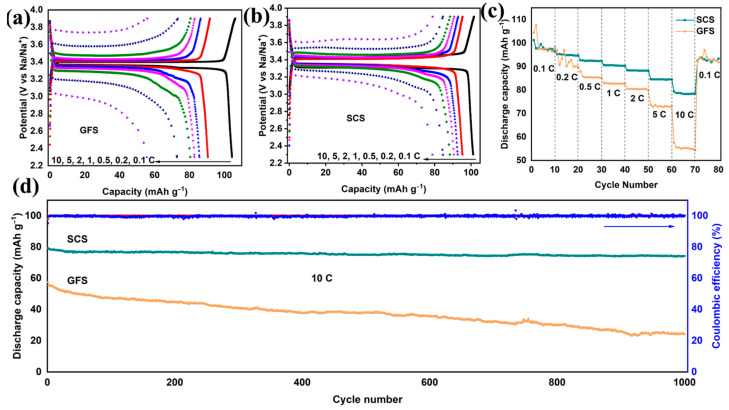
Comparison of electrochemical performances of Na||NVP full cells assembled with SCSs and GFSs. Galvanostatic charging−discharging profile curves of the Na||SCS||NVP (**a**) and Na||GFS||NVP full cells (**b**) from 0.1 C to 10 C. Rate capabilities (**c**) and long-term cycling performances (**d**) of the Na||SCS||NVP and Na||GFS||NVP full cells.

To further demonstrate the application potential of SCSs, the electrochemical performances of Na||NVP full batteries using the SCS and the GFS were tested. As shown in Figure 3a,b, the Na||GFS||NVP and Na||SCS||NVP cells exhibit similar electrochemical reaction potentials (~3.4 V) and discharge capacities (~100 mAh g^−1^) at 0.1 C. When the charge/discharge rate is increased to 10 C, the Na||SCS||NVP cell still displays a discharge capacity of 79.3 mAh g^−1^, which is much higher than that of the Na||GFS||NVP cell (60 mAh g^−1^). It should be noted that the potential hysteresis in the charge−discharge curves of the Na||SCS||NVP cell is only about 1/2 of that of the Na||GFS||NVP cell at 10 C (Figure S2a,b). This phenomenon suggests that Na-ions are able to pass through SCSs more easily and are not over−enriched on the surface of SCSs even at high current densities [40,41]. The rate performance test reveals that the Na||SCS||NVP cell consistently outperforms the Na||GFS||NVP cell in terms of discharge capacity at 0.5−10 C, and the higher the rate, the more pronounced this advantage becomes (Figure 3c). Furthermore, the Na||SCS||NVP cell also exhibits better cycling performance than the Na||GFS||NVP cell at 10 C (Figure 3d). The discharge capacity of the Na||SCS||NVP cell remains 74.2 mAh g^−1^ after 1000 cycles, corresponding to a capacity retention rate of 93.6%. In contrast, the discharge capacity and capacity retention rate of the Na||GFS||NVP cell are only 24.2 and 42.1%, respectively. Moreover, the discharge medium voltage of the full cell using the SCS gradually increases with cycling until it stabilizes near 3.2 V, while that of the full cell using the GFS keeps decreasing (Figure S2c).

In order to understand the chemical stability of SCSs during electrochemical reactions, cyclic voltammetry (CV) tests of both the Na||SCS||NVP and Na||GFS||NVP full cells were executed. Compared with the Na||GFS||NVP cell, the Na||SCS||NVP cell also exhibits only a pair of V^3+^/V^4+^ redox peaks attributed to NVP cathode materials (Figure S3a), which implies that the SCS is in a chemically stable state during electrochemical processes. Electrochemical impedance spectroscopy (EIS) is an efficient testing technique to analyze the internal resistance distribution of a battery. The Na||SCS||NVP and the Na||GFS||NVP share the same EIS graphic characteristics (Figure S3b), i.e., both consist of a semicircle in the high-frequency region and a diagonal line in the low−frequency region. The semicircle is mainly associated with the *R*_ct_ [42]. The Na||SCS||NVP cell displays a smaller semicircle diameter than the Na||GFS||NVP cell, indicating that the former has smaller values of *R*_ct_ than the latter, which implies superior transport kinetics of Na-ions in the former [43]. In addition to this, the diffusion coefficient of Na-ions can be quantitatively calculated using the following equation:

$$D_{Na+}=\frac{R^{2}T^{2}}{{2A}^{2}n^{4}F^{4}C^{2}\sigma^{2}}$$

Since the parameters *R* (gas constant, 8.314 J mol^−1^ K^−1^), *T* (room temperature, 298.15 K), *A* (area of the electrode, 1.13 cm^2^), *n* (the number of electrons involved in electrochemical reactions, 2), *F* (Faraday constant, 96485.4 C mol^−1^) and *C* (the concentration of Na-ions in the unit cell volume, 6.92 × 10^−23^ mol cm^−3^) in the above equations are the same for both the GFS and SCS cells, the Na-ion transport efficiency of these two cells should be inversely related to the *σ* value. By fitting −*Z’*−*ω*^−1/2^ plots to the straight lines in the low-frequency region of the EIS curves (Figure S3c), it is found that the *σ*−values for cells using SCSs and GFSs are 684.2 and 740.1 Ω s^−0.5^ (see Table S4 for details), respectively, which implies a higher *D*_Na+_ in the cell using SCSs.

### 2.2. Mechanical Properties and Functional Group Characterization

**Figure 4:**
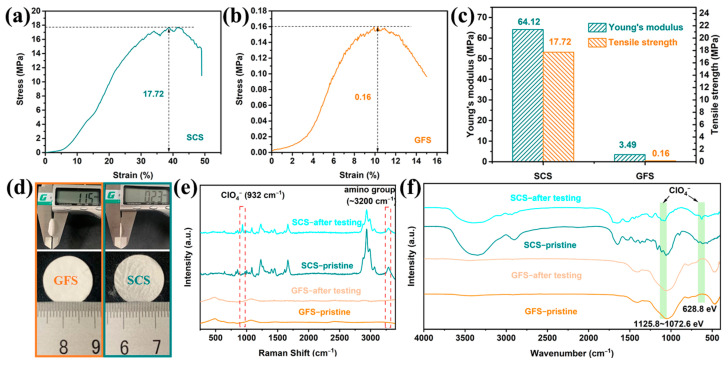
Characterization of mechanical properties and functional groups of the SCS and GFS. Stress–strain curves of the GFS (**a**) and the SCS (**b**). (**c**) Tensile mechanical properties (Young’s modulus, strength). (**d**) Comparison of the appearance and thickness of the SCS and the GFS. (**e**) Raman and (**f**) FT-IR spectra of the SCS and the GFS before and after electrochemical reaction.

The mechanical properties of the separator are not only related to the safety of the battery, but also to whether it can match the winding process for industrial production of the battery. As shown in Figure 4b, the maximum tensile strength of the GFS is only 0.16 MPa which is much lower than the minimum tensile strength value (6.9 MPa) in the winding process, and therefore, the GFS cannot be applied to commercialized column batteries [44]. By comparison, the SCS exhibits a much higher tensile strength (17.72 MPa, Figure 4a) and Young’s modulus (64.12 MPa, Figure 4c). In terms of mechanical properties, the SCS has met the basic requirements for commercialized separators.

The appearance characteristics of the GFS and SCS are shown in Figure 4d, and the flatness of the SCS is slightly worse than that of the GFS, which is due to the slight shrinkage of the sample during the drying process of the SCS. The thicknesses of the SCS and GFS are 0.23 ± 0.01 mm and 1.15 ± 0.02 mm, respectively, and the SCS is clearly more advantageous in the demand for a thinner and lighter separator. Raman and FT−IR spectroscopy were performed on the SCS before and after charging and discharging, respectively. As shown in Figure 4e, a characteristic peak is present at around 3200 cm^−1^ for both SCS samples, which could be attributed to the amino group upon careful comparison with the literature [45]. In addition, the SCS sample after electrochemical testing also exhibits a characteristic peak belonging to ClO_4_^−^ at 932 cm^−1^ that is not found in any other samples [46], suggesting that the SCS sample may anchor the ClO_4_^−^ anion in the electrolyte during the electrochemical reaction. Two sets of characteristic peaks belonging to ClO_4_^−^ at 628.8 and 1125–1072 cm^−1^ are also observed in the FT-IR spectrum of the SCS after the electrochemical test, reconfirming the inference of a possible interaction between silkworm and ClO_4_^−^ (Figure 4f) [47,48].

**Figure 5:**
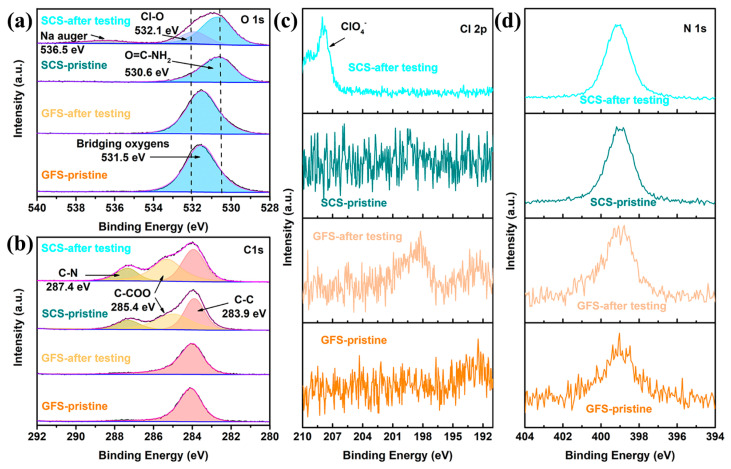
(**a**) O 1s, (**b**) C 1s, (**c**) Cl 2p and (**d**) N 1s XPS spectra of the GFS and SCS samples.

It is noteworthy that the XPS spectra also reveal not only the ability of the SCS sample to interact with ClO_4_^−^ but also the presence of amino groups in the silkworm. As shown in Figure 5a, the SCS and GFS exhibit completely different O1s spectra. Specifically, the O1s spectra of the GFS samples before and after the electrochemical reaction did not change significantly, and only one characteristic peak of bridging oxygen was found at 531.5 eV [49]. Meanwhile, the SCS sample has the characteristic peak representing O=C−NH_2_ at 530.6 eV [50], and the characteristic peaks representing the Cl−O (532.1 eV) bond and Na−auger (536.5 eV) are added to the electrochemically reacted SCS sample [51,52]. Combined with the Cl 2p spectrum (Figure 5c), the presence of a ClO_4_^−^ group in the electrochemically reacted SCS sample can be determined [53]. For the C 1s spectra (Figure 5b), three characteristic peaks are observed for the SCS whether it undergoes electrochemical reaction or not, which correspond to a C−C bond at 283.9 eV, a C−COO bond at 285.4 eV and a C−N bond at 287.4 eV [54,55,56]. In addition, although all samples responded to N 1s spectral detection, the low signal-to-noise ratio of the N 1s spectra of the two GFS samples implies that these samples contain less elemental nitrogen. The high signal-to-noise ratio and good symmetry of the characteristic peaks of the N 1s spectra of the SCS samples indicate that these samples have a high content of nitrogen. The high similarity in the shape and position of the N 1s peaks suggests that the electrochemical reaction does not significantly change the valence state of elemental N.

## 3. Discussion

The low nucleation over-potential of 0.065 V (Figure S1) and the stable cycling of 427 h under test conditions of 0.5 mA cm^−2^–0.5 mAh cm^−2^ (Figure 2b) demonstrate that the Na-ion deposition/dissolution in SCS-assembled cells is not only easier but also has excellent reversibility [57]. In addition to this, the small charge transfer resistance (Figure S3b) implies excellent transport kinetics of Na-ions in the cell using the SCS [58]. Under the synergistic effect of excellent Na-ion transport and deposition/dissolution kinetics, the batteries using SCSs exhibit superior high-rate cycling performance. Therefore, the Na||SCS||NVP full battery displays an initial capacity of 79.3 mAh g^−1^ at 10 C and a capacity retention of 93.6% after 1000 cycles (Figure 3d), which far exceeds the 57.5 mAh g^−1^ and 42.1% of the Na||GFS||NVP full battery. There is a significant effect of separator thickness on the electrochemical performance and that the thickness of the SCS in this work is much lower than that of the GFS (Figure 4d). In order to clarify the specific effect of separator thickness on electrochemical performance, the commercial GFA separator (only 0.34 ± 0.01 mm in thickness), which is thinner than the GFS, was used for a comparison experiment. As shown in Figure S4, the Na||GFA||NVP cell experiences a rapid capacity decline in the multifold test, with the capacity decaying from 115 mAh g^−1^ at 0.1 C to 44 mAh g^−1^ at 5 C until the cell fails at 10 C. The results of this test show that the thinner the separator, the harder it is to obtain excellent electrochemical performance.

**Figure 6:**
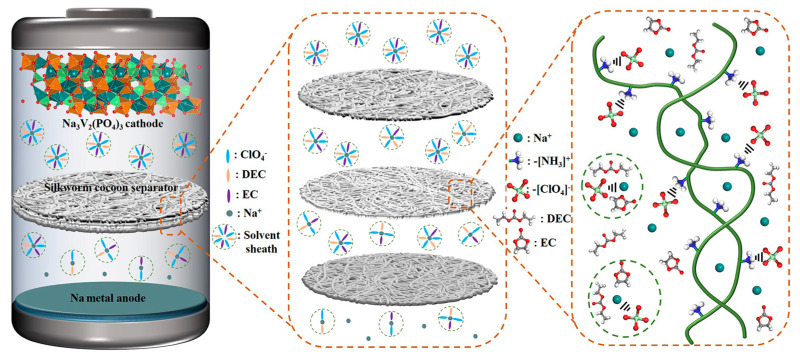
Schematic diagram of the cell structure and the SCS’ action mechanism.

Characteristic peaks belonging to ClO_4_^−^ are observed in the Raman (Figure 4e), FT-IR (Figure 4f) and XPS patterns (Figure 5a,c) of the SCS samples after electrochemical testing, which are not observed in the pristine SCS or the GFS before and after electrochemical testing, suggesting that ClO_4_^−^ could be firmly attached to the SCS fibers. Combined with the previous literature, it can be reasonable to assume that this phenomenon is most likely caused by cationic functional groups (such as amino groups) on the protein molecule [59,60]. From the conclusions of previous studies, it is known that Na-ions undergo solvation reactions with anions and solvents during transport in the electrolyte [61,62]. The solvated Na-ions increase in both mass and volume, which undoubtedly reduces the transport kinetics of the Na-ions [63]. As shown in Figure 6, when solvated Na-ions pass through the SCS, the amino functional groups on silkworm proteins disrupt the solvent sheath by anchoring ClO_4_^−^, thereby releasing free Na−ions. As a result, the transference number (*T*_Na+_ = 0.81), transport kinetics and deposition/solvent properties of Na-ions are improved. On this basis, the advantages in mechanical strength and thinness make SCSs more promising than GFSs for commercialized sodium batteries.

## 4. Materials and Methods

### 4.1. Fabricating SCS

**Figure 7:**
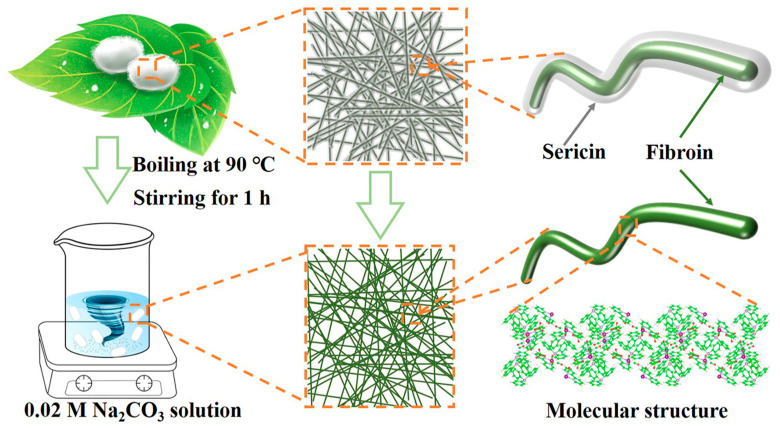
Schematic diagram of the preparation process of silkworm cocoon separator.

The SCS fabrication process is shown in Figure 7. First, 0.04 mol Na_2_CO_3_ (99.8%, Shanghai Macklin Biochemical Technology Co., Ltd., Shanghai, China) was dissolved in 2 L deionized water to prepare the washing solution and heated to boiling. The mulberry silkworm cocoons (Beijing Tong Ren Tang group Co., Ltd., Beijing, China) were then poured into the boiling liquid and treated for 20 min to remove the sericin. Next, the cocoons were fished out, washed three times with deionized water and exfoliated of their inner layers. Subsequently, the inner layer of the cocoon was flattened, sandwiched between two glass plates to fix the shape, and transferred to a vacuum oven at 80 °C until dry. The dried cocoons’ inner layer was cut into 16 mm discs to be used as separators, which were named SCS. Thick (Whatman GF/D) and thin (Whatman GF/A) glass fiber separators were used as comparison samples, named GFS and GFA, respectively.

### 4.2. Material Characterization

The microscopic morphology of the SCS, GFS and sodium metal anode was observed by field-emission scanning electron microscopy (FESEM, Thermo Fisher Scientific FIB-SEM GX4, Waltham, MA, USA). The XRD data were measured by a Bruker D8 diffractometer at a sweep speed of 0.3° s^−1^ over a range of 5–80° with Cu Kα (λ = 1.5418 Å). FT-IR absorption spectroscopy (Thermo Scientific Nicolet iS50, Waltham, MA, USA) was used to test the functional groups of different separators in the range of 600 cm^−1^~4000 cm^−1^; all samples were washed three times in water under sonication for 10 min before testing. An electronic universal testing machine was employed to test the mechanical properties of different separators. An X-ray photoelectron spectrometer (ThermoFischer, ESCALAB 250Xi, Waltham, MA, USA) was used in this experiment for XPS tests. In this case, the vacuum of the analysis chamber was 2 × l0^−8^ Pa, the excitation source was an Al Kα ray (hυ = 1486.6 eV) and the operating voltage was 12.5 kV. Raman spectroscopy tests were performed on a ThermoFischer Dxr3xi Raman microscopy (Waltham, MA, USA) instrument equipped with an Ar^+^ laser (λ = 532 nm).

### 4.3. Electrochemical Measurements

All the electrochemical performance tests were performed based on CR2025-type coin batteries, and the battery assembly was completed in an argon-filled glovebox (H_2_O < 0.1 ppm, O_2_ < 0.1 ppm, Mikrouna, Shanghai, China). For the preparation of the NVP cathode, NVP (Guangdong Canrd New Energy Technology Co., Ltd., Dongguan, China), polyvinylidene fluoride (PVDF) and acetylene black with a mass ratio of 8:1:1 were dispersed in N-methyl-2-pyrrolidine (NMP) to form a uniform slurry and then coated on aluminum foils. After they were dried in a vacuum oven at 110 °C for 10 h, these aluminum foils were cut into discs with a diameter of 14 mm to be the NVP cathode. An amount of 50 μL of electrolytes (1 M NaClO_4_ dissolved in EC:DEC=1:1 (*v*/*v*) with 5% FEC) was used for each battery. All batteries used Na foil (~100 μm) as the anode, and the difference was that the working electrodes were NVP, Cu foil or Na foil, with the GFS or SCS as the separator. The galvanostatic charging–discharging, over-potential and cycling performance of the batteries were measured on a Land BT 2000 battery test systems at 25 °C. The potentiostatic modal EIS (frequency range from 10^−2^ to 10^5^ Hz with an amplitude value of 10 mV), CV (voltage range from 2.3 to 3.9 V with a scanning rate of 0.1 mV s^−1^) and CA (open-circuit voltage used as initial voltage, executed for 1000 s at 10 mV bias) curves were tested on a CHI660e electrochemical workstation.

## 5. Conclusions

In summary, we developed a novel natural SCS for sodium metal batteries. Systematic structural characterization reveals that the cationic functional groups (such as amino groups) enriched in the SCS samples can anchor the ClO_4_^−^ solvent sheath around the Na-ions, realizing a high Na^+^ transference number (T_Na+_ = 0.81) and substantially enhancing the transport kinetics. Benefiting from this, the Na||SCS||Na symmetric cell can be stably cycled for over 400 h at 0.5 mA cm^−2^–0.5 mAh cm^−2^. Moreover, the Na||SCS||NVP full battery displays a reversible discharge specific capacity of 79.3 mAh g^−1^ at 10 C and a remaining capacity of up to 74.2 mAh g^−1^ after 1000 cycles. In addition, the mechanical properties and thickness of the SCS are also completely superior to those of the GFS, which means that the SCS has more potential for commercial application than the GFS.

## Data Availability

The data that support the findings of this study are available from the corresponding author upon reasonable request.

## Supplementary Materials

The following supporting information can be downloaded at https://www.mdpi.com/article/10.3390/molecules29204813/s1: Figure S1: Voltage-capacity curves of Cu||SCS||Na and Cu||GFS||Na asymmetric cell at a current density of 0.5 mA cm^−2^; Figure S2: Charge-discharge voltage profiles for Na||NVP full batteries using GFS (a) and SCS (b) separator. The discharge medium voltage at different cycles of Na||GFS||NVP and Na||SCS||NVP full batteries; Figure S3: (a) CV curves at a scan rate of 0.1 mV s^−1^, (b) EIS curves (inset: equivalent circuit diagram) and (c) relationship plots of the impedance as a function of the inverse square root of angular frequency in a low-frequency region of Na||SCS||NVP and Na||GFS||NVP full cells; Figure S4: Galvanostatic charging-discharging profile curves of Na||GFA||NVP; Table S1: Comparative table of electrochemical properties of cocoon based separators; Table S2: Summary table of structural parameters of SCS sample; Table S3: Parameters reported for EIS curves in Figure 2c,d fitted by equivalent circuits. Table S4: Parameters reported of the EIS curves in Figure S3 after equivalent circuits and linear fitting.
