# Supplementary material for: Natural Silkworm Cocoon-Derived Separator with Na-Ion De-Solvated Function for Sodium Metal Batteries
Source: Molecules. 2024 Oct 11;29(20):4813. doi: 10.3390/molecules29204813 (PMC11510509; doi:10.3390/molecules29204813)
Supplement: Supplementary file 1 [file molecules-29-04813-s001.zip › molecules-3200474-supplementary.pdf]

Natural silkworm cocoon derived separator with Na-ion de-solvated function for sodium-metal batteries

Zhaoyang Wang [1], Zihan Zhou [1], Xing Gao [1], Qian Liu [1], Jianzong Man [1], Fanghui Du [1] and Fangyu Xiong [2,3]*

[1] Shandong Provincial Key Laboratory of Chemical Energy Storage and Novel Cell Technology, College of Chemistry Engineering, School of Physics Science and Information Technology, Liaocheng University, Liaocheng, 252059, P.R. China
[2] College of Materials Science and Engineering, Chongqing University, Chongqing, 400044, China
[3] Chongqing Institute of New Energy Storage Materials and Equipment, Chongqing 401135, China
* Correspondence: xfy@cqu.edu.cn

Keywords: sodium metal battery; separator materials; natural silkworm cocoon; Na^+-ion de-solvated function; mechanistic analysis

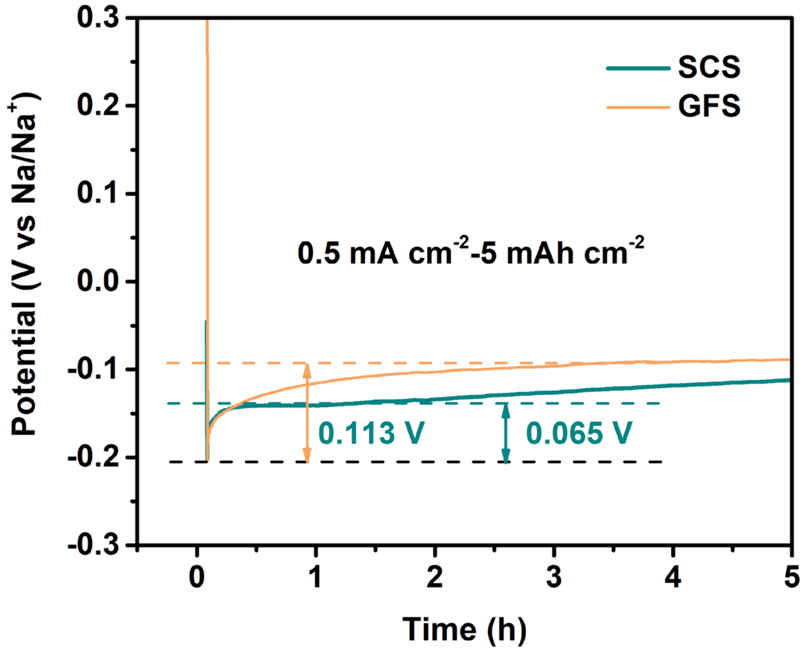

Figure S1. Voltage-capacity curves of Cu||SCS||Na and Cu||GFS||Na asymmetric cell at a current density of 0.5 mA cm^{-2}.

Table S1. Comparative table of electrochemical properties of cocoon based separators.

Silk cocoon treatment methods	Treatment effect	Battery type	Cathode material	Electrolyte composition	High rate performance (mAh g^{-1}/C)	Cycle performance (mAh g^{-1}/C/cycle number)	Ref.
-	-	LIBs	$LiFePO_4$	[Emim]TFSI	~15/1	8/1/50	17
-	-	LIBs	$LiFePO_4$	1 M $LiPF_6$ in EC:DMC (1:1 vol.)	~106.2/1	86/1/50	17
Stacking layers	Constructing gradient layers	LIBs	$LiFePO_4$	1 M $LiPF_6$ in EC:EMC: DMC (1:1:1 vol.)	~130/1	116/1/100	18
Salt leaching	Manufacturing pore structure	LIBs	$LiFePO_4$	1 M $LiPF_6$ in EC:DMC (1:1 vol.)	~66.9/2	56.9/2/55	19
Salt leaching	Manufacturing pore structure	LIBs	$LiFePO_4$	1 M $LiPF_6$ in EC:DMC (1:1 vol.)	148/2	85/2/100	20
Lyophilization	Manufacturing into sponges	LIBs	$LiFePO_4$	1 M $LiPF_6$ in EC:DMC (1:1 vol.)	99/2	~76/2/50	21
O_2 plasma	Increasing fiber roughness and oxygenated functional groups	LIBs	$LiFePO_4$	1 M $LiPF_6$ in EC:DMC (1:1 vol.)	96.7/5	44.4/5/55	22
Boiling	Removing sericin	SMBs	$Na_3V_2(PO_4)_3$	1 M $NaClO_4$ in EC:DEC (1:1 vol.)	79.3/10	74.2/10/1000	This work

Footnote: [Emim]TFSI: 1-ethyl-3-methylimidazolium bis(trifluoromethylsulfonyl)imide; EC: ethylene carbonate; DMC: dimethyl carbonate; EMC: ethyl methyl carbonate; DEC: diethyl carbonate.

Table S2. Summary table of structural parameters of SCS sample.

Index	2θ (°)	FWHM	Area (%)	D (nm)
(100)	9.1	4.15	10.51	1.93
(210)	20.5	3.74	51.08	2.25
(002)	23.0	6.53	37.34	1.31
(300)	29.4	2.15	1.08	4.15

Footnotes: 2θ: angle of diffraction; FWHM: half-peak width; D: Average thickness of grains in the direction perpendicular to the grain surface.

Table S3. Prameters reported for EIS curves fitted by equivalent circuits.

Sample	R_s (Ω)	*CPE1* (S sec^n)	R_{ct} (Ω)	*CPE2* (S sec^n)	R_{CPE} (Ω)	C_{dl} (F)	R_{dl} (Ω)	Measure errors in Z	Chi-squared
**GFS-Initial**	4.9	2.2×10^{-6}	205.7	1.7×10^{-4}	42.1	3.0×10^{-6}	0.8	<4.1%	1.7×10^{-3}
**GFS-Final**	4.9	2.2×10^{-6}	194.2	4.6×10^{-5}	55.0	8.9×10^{-6}	1180	<3.8%	1.5×10^{-3}
**SCS-Initial**	8.0	8.3×10^{-6}	134.6	4.6×10^{-4}	980	1.3×10^{-5}	975.9	<2.1%	4.4×10^{-4}
**SCS-Final**	7.9	8.2×10^{-6}	129.8	3.0×10^{-4}	1107	1.2×10^{-5}	1043	<2.1%	4.5×10^{-4}

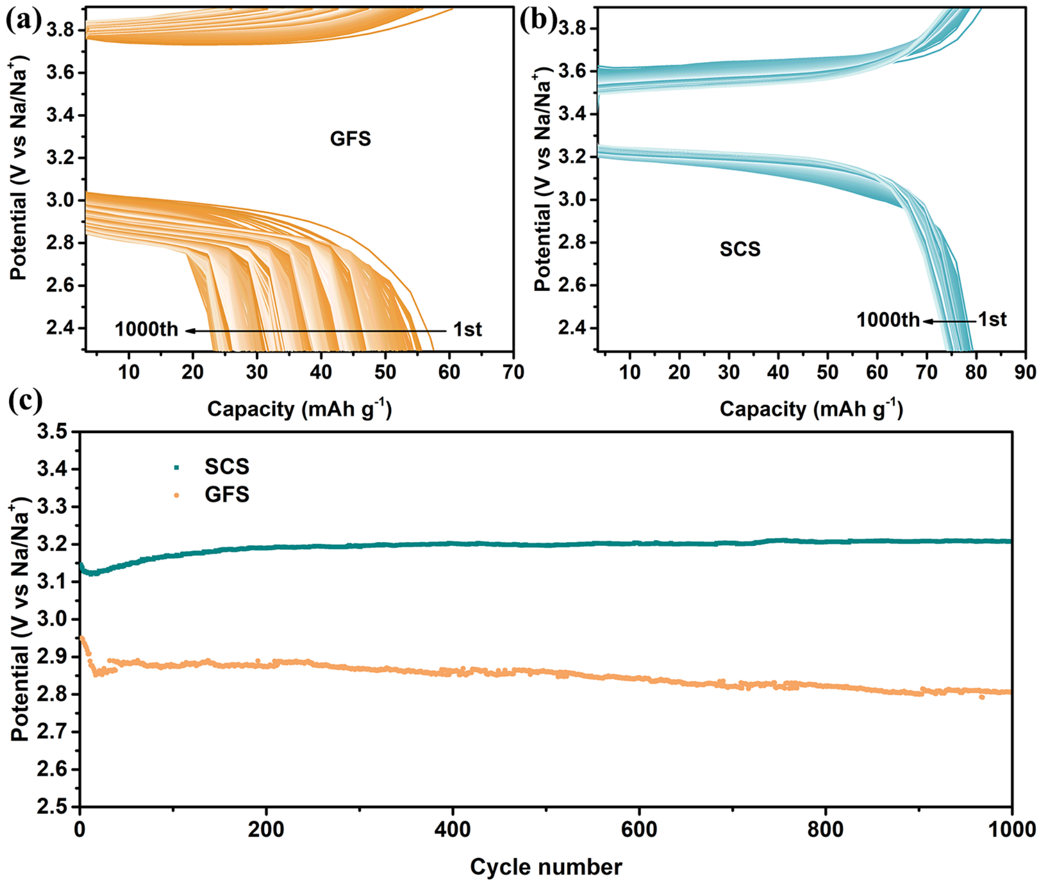

Figure S2. Charge-discharge voltage profiles for Na||NVP full batteries using GFS (a) and SCS (b) separator. The discharge medium voltage at different cycles of Na||GFS||NVP and Na||SCS||NVP full batteries.

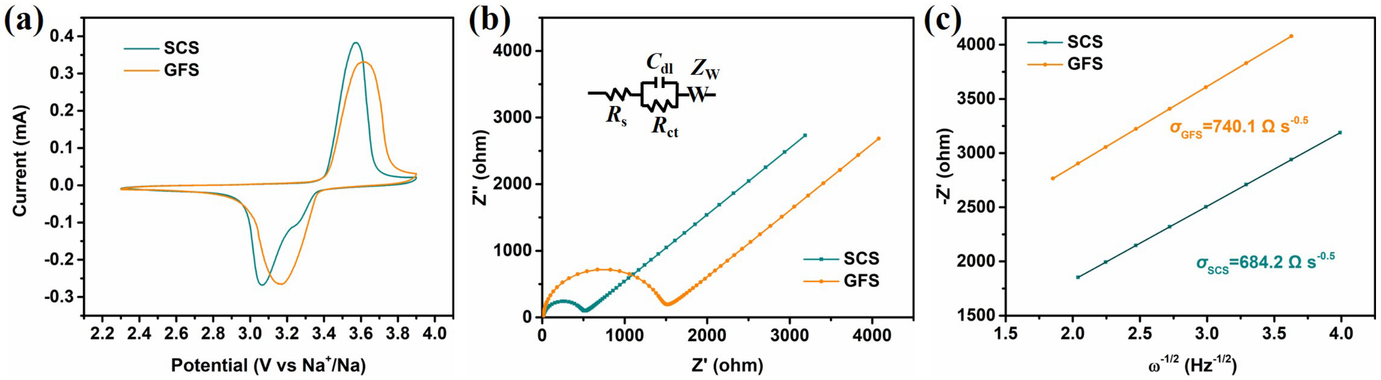

Figure S3. (a) CV curves at a scan rate of 0.1 mV s^{-1}, (b) EIS curves (inset: equivalent circuit diagram) and (c) relationship plots of the impedance as a function of the inverse square root of angular frequency in a low-frequency region of Na||SCS||NVP and Na||GFS||NVP full cells.

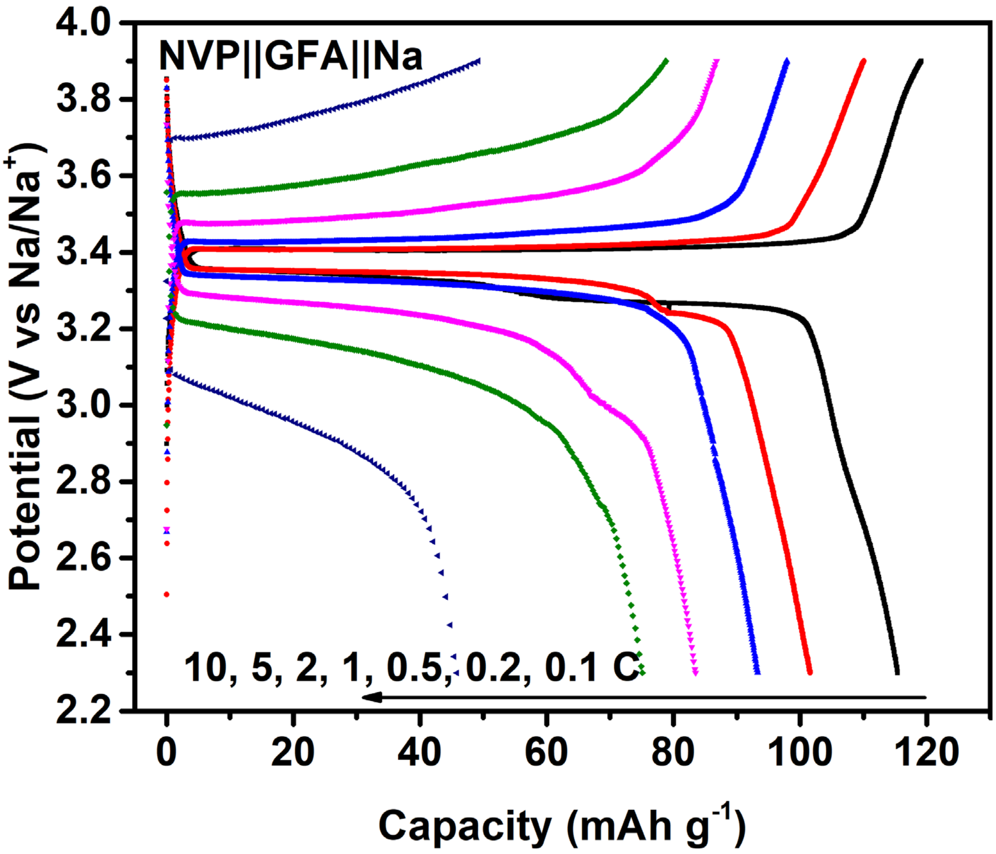

Figure S4. Galvanostatic charging-discharging profile curves of Na||GFA||NVP.

Table S4. Parameters reported of the EIS curves in Figure S3 after equivalent circuits and linear fitting.

Sample	R_s (Ω)	C_{dl} (F)	R_{ct} (Ω)	σ (Ω $s^{-0.5}$)	Measure errors in Z	Chi-squared	Linear fitting residual sum of squares
**GFS**	9.76	1.0×10^{-6}	1385.0	740.1	<6.1%	4.2×10^{-2}	5.1×10^{-7}
**SCS**	16.27	2.1×10^{-6}	378.3	684.2	<6.8%	4.9×10^{-2}	3.3×10^{-7}
